# Diagnostic specificity of the child psychosis-risk screening system with a focus on the differentiation of schizophrenia spectrum disorders and neurodevelopmental disorders

**DOI:** 10.3389/frcha.2023.1230346

**Published:** 2023-08-04

**Authors:** Yukiko Hamasaki, Yuko Sakaue, Masahiro Matsuo, Riku Sanada, Takao Nakayama, Shugo Michikoshi, Satoko Ueba, Naoki Kurimoto, Takatoshi Hikida, Toshiya Murai

**Affiliations:** ^1^ Faculty of Contemporary Society, Kyoto Women’s University, Kyoto, Japan; ^2^ Department of Psychiatry, Shigasato Hospital, Otsu, Japan; ^3^ Department of Pediatrics, Shiga University of Medical Science, Otsu, Japan; ^4^ Department of Psychiatry, Shiga University of Medical Science, Otsu, Japan; ^5^ Faculty of Data Science, Kyoto Women’s University, Kyoto, Japan; ^6^ Department of Pediatrics, Saiseikai Moriyama Municipal Hospital, Moriyama, Japan; ^7^ Laboratory for Advanced Brain Functions, Institute for Protein Research, Osaka University, Suita, Japan; ^8^ Department of Psychiatry, Kyoto University Graduate School of Medicine, Kyoto, Japan

**Keywords:** schizophrenia, autism spectrum disorder, neurodevelopmental disorders, child psychosis-risk screening system, child behavior checklist, risk screening

## Abstract

**Background:**

Research on early psychosis has begun to identify psychiatric characteristics of the prodromal period of schizophrenia; however, subclinical characteristics of children in non-psychiatric fields have not been fully investigated. In our previous study, we developed the Child Psychosis-risk Screening System (CPSS).

**Objective:**

In the present cross-sectional study, we attempted to identify the risk of developing psychosis in pediatric (*n* = 216) and psychiatric outpatients (*n* = 120), aged 6– 18 years, with the CPSS.

**Methods:**

An analysis of variance of CPSS risk was performed in six diagnostic categories to examine specificity for each diagnosis. Receiver operating characteristic (ROC) curve analysis was conducted using the onset of schizophrenia spectrum as the outcome, and the discriminatory power and cut off values of the CPSS were determined. Logistic regression analysis was performed using clinical data to identify factors associated with the risk group (those at high risk of developing psychosis in the future) identified using the CPSS.

**Results:**

There were significant differences in risk variance among diagnostic categories (*p *< 0.001), especially between schizophrenia spectrum disorders (SSD) and neurodevelopmental disorders (*p *= 0.001). CPSS had sufficient discriminatory power for SSD diagnosis [area under the ROC curve = 0.853 (95% confidence interval: 0.774–0.931)]. The cut off value for the risk of SSD was determined to be 98.1%, achieving the best mean of the sum of sensitivity (90.9%) and specificity (84.0%). Cross-sectional logistic regression analysis showed that along with “SSD diagnosis,” “winter birth,” and “maltreatment” were factors associated with the risk group (odds ratio = 38.05 [*p *= 0.001], 2.30 [*p *= 0.016], and 0.12 [*p *= 0.024], respectively).

**Conclusion:**

CPSS may have potential use in the early detection of psychosis and differentiation from neurodevelopmental disorders, but this study was small and further studies with larger sample sizes and longitudinal study designs are required prior to its use in routine clinical practice.

## 1. Introduction

Since the neurodevelopmental hypothesis of schizophrenia was proposed in the 1980s (1, 2), increasing evidence has accumulated to support it (3). The early neurodevelopmental model identifies the factors of the traditional vulnerability model (4), and is a multi-domain model that describes the interaction between genes and epigenetic as well as environmental triggers (such as infection and/or trauma, hypoxia, malnutrition, and childhood neighborhood disadvantage) in the etiology of schizophrenia (5, 6).

The clinical manifestation of vulnerability is observed in the prodromal phase, generally during adolescence and young adulthood (7, 8). The prodrome is defined as a group of symptoms that is not yet specific to schizophrenia but indicates a continuous transition to the disorder (9). Huber identified impaired social functioning and cognitive problems as basic symptoms of schizophrenia (10). The prodromal phase is significant in terms of application of early interventions in psychosis and is considered a “window of opportunity” to reduce the transition to psychosis and to inhibit its progression (6).

The period preceding the prodromal phase, when the signs are in the subclinical range, is called the premorbid stage. Prospective studies such as the familial high risk and birth cohort studies have indicated that common characteristics of the early-childhood premorbid stage of schizophrenia include isolated tendencies, poor social functioning, and developmental delays in motor and language milestones (11–15). The aforementioned features are found to be stable during the developmental stages, and their continuity to disease indicates the possibility that they can be considered as the first manifestation of prodrome (16). Considering the early neurodevelopmental disorder hypothesis, it is logical and natural to assume that there is a continuum of signs in the premorbid stage. This makes it difficult to outline a boundary between the premorbid stage and prodrome phase, and the prodrome may need to be set up much earlier than that previously envisioned. It is not uncommon for symptoms to be subclinical in psychiatry, but already within the clinical domain in pediatrics. The earlier we can identify the risk of psychosis in pediatric cases and intervene, the earlier we can open the “window of opportunity”.

The paradigm known as Clinical High Risk of developing Psychosis (CHR-P), associated with the goal of providing timely preventive interventions to those at high-risk of developing psychosis, is still in its infancy from a pediatric perspective and is far from complete in its identification of psychosis risk in children (17). In recent years, however, several challenging studies have attempted to predict the prognosis of children in the premorbid stage (18–22). A systematic review of longitudinal studies examined the pre-onset cognitive impairments and their timed appearances in schizophrenia spectrum disorders (SSD) and found that impairments start early in life, in line with the neurodevelopmental hypothesis of schizophrenia (15).

As there are several prognostic markers, and as correlations exist between these markers and emotional/behavioral problems in childhood, it is reasonable to assume that the risk of developing psychosis can be screened early by assessing the child's emotional/behavioral characteristics. In clinical practice, it is desirable to screen children who do not yet have psychiatric symptoms but who have been seeing a pediatrician for a long period of time because of psychosomatic symptoms or adjustment problems and who would benefit from early prevention of and intervention for psychosis (23, 24).

Researchers have long been searching for a tool to identify risk factors for psychosis (25, 26). To date, no childhood psychosis risk identification tool exists, although clinical tools have been developed to identify psychosis risk, all of which are were designed for use in adolescents and adults. The Child Behavior Checklist (CBCL) has been utilized in studies of children to identify correlates between behavioral problems in early childhood and later risk of schizophrenia (27–30). In our previous study, we conducted a retrospective survey using the CBCL/4-18 for schizophrenia patients in their 20s to extract data on their subclinical characteristic patterns during childhood (30). We used the CBCL because it is an exhaustive checklist, and we considered it would be effective in extracting multidimensional patterns of a child's early signs. Our previous study using the CBCL revealed that, for adult patients with schizophrenia, psycho-behavioral characteristics such as withdrawal, thought problems, and lack of aggression were already present in childhood (6–8 years), albeit at a subclinical level. Receiver operating characteristic (ROC) curve analysis revealed that the logistic regression model (a pattern of characteristics extracted from logistic regression analysis) discriminates schizophrenia patients/controls with an area under the curve (AUC) of 0.828 [95% confidence interval (CI): 0.762–0.894].

We hypothesized that the “combined patterns of subclinical characteristics” found in the patient population are potential endophenotypic markers of the risk group and developed the Child Psychosis-risk Screening System (CPSS), which uses this logistic regression model with coefficients of the eight syndrome subscales of CBCL (Supplementary Appendix S1) as the algorithm for identifying psychosis risk in children (30). The CPSS calculates the risk% of developing psychosis from the subject's *t*-scores for eight syndromes on an interactive web system. The diagnostic specificity of the CPSS algorithm, when other disorders are included, has not been confirmed, because the previous retrospective study only aimed to identify patients with schizophrenia.

Therefore, in the present study, we aimed to:

(1) Elucidate the diagnostic specificity and discriminative power of the CPSS by measuring risk indicators in pediatric and psychiatric outpatients;
(2) Determine the number of high-risk groups above the cutoff value among the pediatric and psychiatric patients and to explore the factors (e.g., environmental factors) related to the high-risk groups.
(3) Test a risk-predicting algorithm for identifying children who would benefit from early intervention strategies to reduce the risk of developing psychosis in pediatric clinical application.

This study is novel in that it expands the prodromal concept to include children who remain in a subclinical state without psychiatric symptoms.

## 2. Materials and methods

### 2.1. Participants

A total of 336 outpatients aged 6–18 years (216 from pediatric developmental outpatient department and 120 from psychiatric department) were recruited (Supplementary Table S1). In Japan, there are few outpatient clinics specializing in child psychiatry; thus, pediatric developmental outpatient departments are primarily responsible for dealing with children's mental health (including conditions like psychosomatic symptoms, developmental delays, adjustment problems, child abuse). Patients were diagnosed at the clinic by the treating physician and, additionally, as part of the research protocol. All patients were screened with the Structured Clinical Interview for DSM-5-Research Version (SCID-5-RV) (31) and the Structured Clinical Interview for DSM-IV Childhood Diagnoses (Kid-SCID) (32). Inclusion criteria were as follows: (i) patients already diagnosed at the start of the study with a psychiatric condition; (ii) patients that freely and voluntarily provided written or verbal informed assent; and (iii) those whose eligibility was assessed by the treating physician in charge of the research. Exclusion criteria were as follows: (i) patients that were dependent on alcohol or any illicit substances; (ii) those that had acute-state psychosis or moderate or severe intellectual disability, as obtaining informed assent from these patients was difficult; and (iii) those whose parents were unable to complete the questionnaire owing to intellectual disability or a psychotic state.

Outpatients were recruited from a university hospital and district hospitals in the Kansai area between December 2019 and December 2021.

#### 2.1.1. Ethical approval

This study was approved by the Ethics committee of Kyoto Women's University, Japan (approval number: 30-10, 2019) and Shiga University of Medical Science, Japan (R2019-135); it was conducted in accordance with the latest version of the tenets of the Declaration of Helsinki. If the inclusion/exclusion criteria were satisfied, parents were requested to provide written informed consent after receiving a full explanation of the study.

### 2.2. Measures

#### 2.2.1. Measuring psycho-behavioral characteristics using CBCL/6–18

The CBCL is a checklist developed by Achenbach et al. to comprehensively assess children's emotional, behavioral, and physical problems and is to be filled by the parent/guardian for problems between now and the past 6 months (33). The raw scores of the 120 problem items are used to calculate scores on eight syndrome subscales (i.e., anxious/depressed, withdrawn/depressed, somatic complaints, social problems, thought problems, attention problems, rule-breaking behavior, and aggressive behavior). Standard values vary by country, sex, and age (34–36); therefore, scores on the subscales are converted to *t*-scores based on these standard values. To evaluate children's clinical and subclinical characteristics, we administered the new version of CBCL/6–18 (33). One parent or both parents together completed the CBCL at the hospital, thereby resulting in one record per child. The primary difference between the CBCL/4–18 and the CBCL/6–18 is the updated normative data and a change in the lower limit of the age range. If a child's functioning has not changed much between assessments on the old and new versions of a form, the child's syndrome scores should be equivalent to nearly the same percentiles and *t*-scores on each version (33).

#### 2.2.2. Child psychosis-risk screening system

Using the CPSS developed from our previous retrospective study (30), this study attempted to identify the risk of the development of psychosis in pediatric patients. The CPSS calculates a child's risk% from the *t*-scores of the CBCL syndrome subscales. Our previous study used CBCL/4–18, while *t*-scores of CBCL/6–18 were used in the present study. As CBCL/6–18 *t*-scores should be equivalent to *t*-scores of CBCL/4–18, no specific improvements were made to the CPSS algorithm other than the order of *t*-scores to be entered into the algorithm (Supplementary Appendix S2).

#### 2.2.3. Clinical data: sex, age, winter birth, chief complaint, diagnosis, maltreatment, bullying, and withdrawal

Data on demographics (age, sex, winter birth); chief complaint; clinical diagnosis according to DSM-5; and information on abuse, bullying, and withdrawal were collected by the attending physicians who were research collaborators. Patients’ diagnoses were classified into six major categories i.e., Neurodevelopmental Disorders, SSD, Depressive Disorders, Anxiety Disorders (including Obsessive-Compulsive Disorder), Somatic Symptom Disorders, and Others. Winter birth was defined as December–February birth (*n* = 109). The chief complaint was divided into two categories: physical (*n* = 58) and non-physical (*n* = 278); those in the former group attended clinic with a primarily physical symptom rather than a non-physical symptom, which is commonly seen in pediatric practice. Diagnoses of the pediatric and psychiatric patients are shown in Supplementary Table S2. Maltreatment, bullying, and withdrawal were assessed using our original ad-hoc scales. Maltreatment was divided into two categories: with (*n* = 47) and without (*n* = 289) maltreatment. Bullying victimization was rated on a 4-point scale (0: not at all applicable, 1: sometimes applicable, 2: often applicable, 3: always applicable). Withdrawal was rated on a 4-point scale [0: not at all applicable (rarely misses school), 1: sometimes applicable (sometimes misses school), 2: often applicable (misses school more than half a month and rarely goes out), 3: always applicable (never goes to school and rarely leaves home for more than 6 months)].

### 2.3. Statistical analyses

When CPSS risk of the participants was identified, the Shapiro–Wilk test was used to determine if the data were normally distributed. Mean CPSS risk% and mean logit(*p*) were calculated for each diagnosis and chief complaint (physical and non-physical). The mean values in the normal control group were obtained from our previous retrospective study data (30).

To evaluate the specificity of CPSS risk by diagnosis, an analysis of variance of CPSS risk by diagnostic category was performed. The Kruskal–Wallis test was employed in the analysis of variance to examine whether there were differences in variance between categories. Multiple comparisons using the Bonferroni method were then performed to test whether there were significant differences between the two groups. Furthermore, after categorizing neurodevelopmental disorders into autism spectrum disorder (ASD), attention deficit hyperactivity disorder (ADHD), and others, a similar analysis of variance was performed to compare these with the CPSS risk for SSD.

To derive the optimal cutoff points for the CPSS, we performed ROC curve analysis, in all 336 study participants, using the confirmation of schizophrenia spectrum diagnosis as the dependent variable (outcome). To determine the predictive ability of the CPSS, the AUC was computed using non-parametric trapezoids. Two groups were created based on the CPSS values: a “risk” group and a “non-risk” group, defined as the group with CPSS values above and below the CPSS cutoff value, respectively. Individuals with CPSS values exceeding the cutoff (i.e., those in the risk group) were considered to be at high risk of developing psychosis in the future. The cutoff value was defined as the point on the ROC curve where the sensitivity and specificity are optimal.

Cross-sectional logistic regression analysis was performed using the aforementioned clinical data to identify factors associated with the risk group identified using the CPSS. Sex, winter birth, maltreatment, physical (i.e., not non-physical) chief complaint, and diagnosis were processed as categorical variables. Diagnostic variables were included in the regression to attempt to see if the presence of these diagnoses contributed to the statistical prediction of whether a participant is in the risk group as identified by the CPSS. Analysis was performed with clinical data as the covariate and the CPSS risk group (i.e., the group exceeding the CPSS cutoff) as the dependent variable. First, a linear regression procedure was performed using the same list of covariates and dependent variables as in the logistic regression to diagnose multicollinearity. Multicollinearity was tested using the variance inflation factor (VIF) statistic; variables with VIF >3.0 were considered as having multicollinearity and were excluded from the covariates in the logistic regression analysis. To evaluate goodness-of-fit for the logistic regression model, the Cox–Snell and Nagelkerke R-square value and discrimination accuracy were obtained. The goodness-of-fit test of Hosmer and Lemeshow was also performed. Any between-group difference was estimated as an odds ratio (OR) with a 95% confidence interval (CI).

All analyses were performed using SPSS for Windows (version 22.0; IBM Corporation). In all tests, the level of significance was set at *p *< 0.05. 

## 3. Results

### 3.1. Identification of participants’ risk of psychosis

The mean CPSS risk% of the participants was 52.2% ± 40.6. The risk% had a U-shaped distribution and did not follow a normal distribution (Supplementary Figure S1). Logit transformation of the risk% confirmed that it followed a normal distribution [Shapiro–Wilk test determined (*p*) = 0.429, Supplementary Figure S2]. This logit value [logit(*p*) = *z*, Supplementary Appendix S2] should be used in statistical analysis as a CPSS risk indicator. The mean logit(*p*) for each diagnosis and chief complaint are shown in Supplementary Table S3. There was no significant difference in logit(*p*) (*t *= −0.365, *p *= 0.715) between the physical and non-physical chief complaints.

### 3.2. Specificity of CPSS risk by diagnostic categories

**Figure 1:**
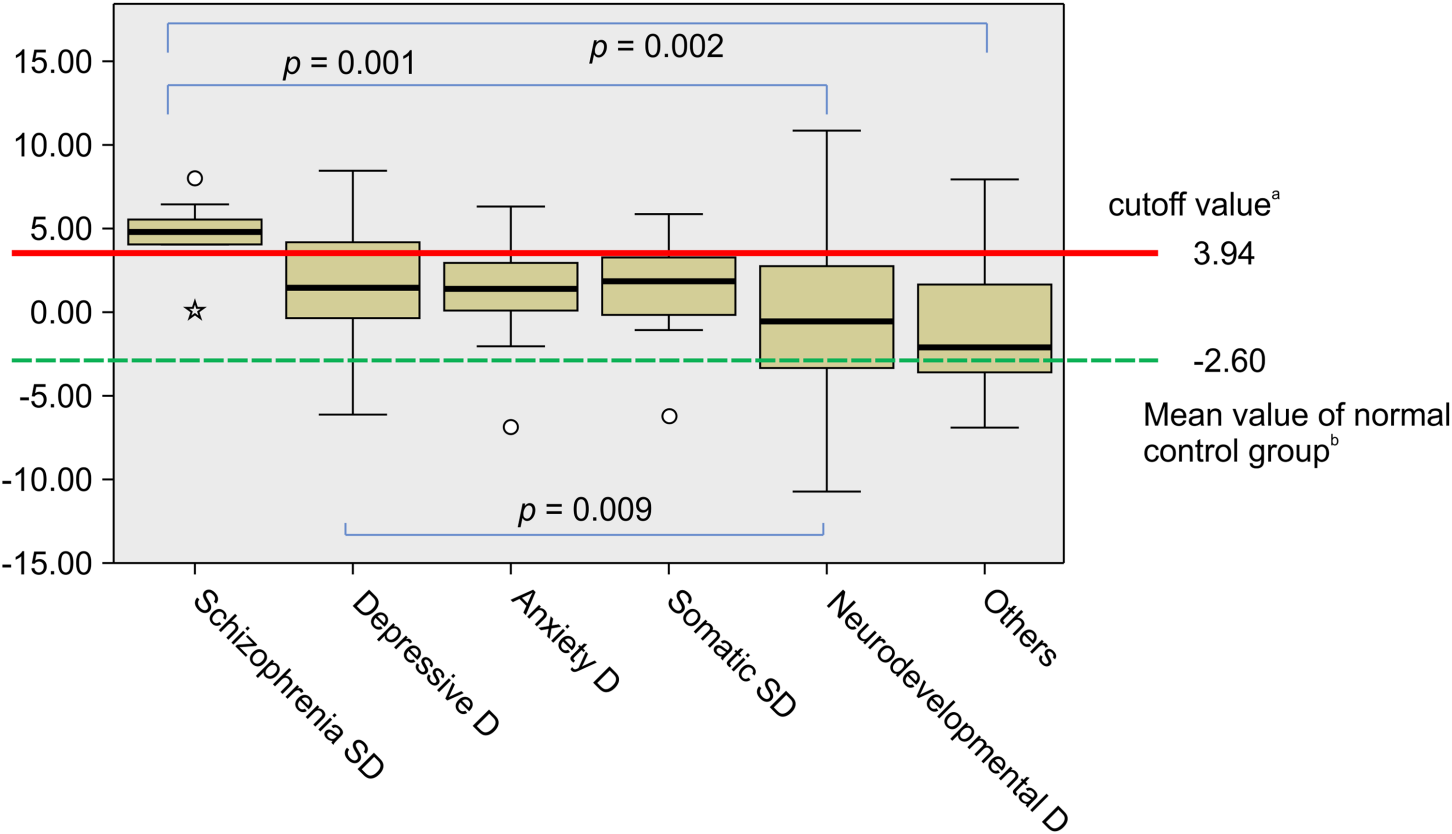
Analysis of variance of CPSS risk for six diagnostic categories: Box-plots of the logit(*p*). ^a^The ROC analysis resulted in a cutoff value of 3.94 for logit(*p*) (sensitivity: 90.9% and specificity: 84.0%). ^b^The mean of logit(*p*) for the normal control group is sourced from our previous retrospective study (30). There was a difference in logit(*p*) among the six diagnostic categories (Kruskal–Wallis test; *p *< 0.001). The increase in logit(*p*) was more marked in patients with schizophrenia spectrum disorders (SSD) than in those with neurodevelopmental disorders (Multiple comparisons using Bonferroni's method; *p *= 0.001). D, disorder; Somatic SD, somatic symptom disorder; ROC, receiver operating characteristic; CPSS, child psychosis-risk screening system.

The mean CPSS risk% and mean logit(*p*) for each of the diagnostic categories are shown in Table 1. The means of the normal control group [sourced from our previous retrospective study (30)] are also shown in Table 1. The mean CPSS risk% in SSDs was 94.59 ± 14.85, with a logit(*p*) of 4.64 ± 1.93, which was markedly higher than those in other diagnostic categories. Analysis of variance revealed significant differences in risk [logit(*p*)] variance between the six diagnostic categories (Kruskal–Wallis test; *p *< 0.001), particularly between SSD and neurodevelopmental disorder groups (multiple comparison using Bonferroni's method; *p *= 0.001). The results of the analysis of variance are shown in Figure 1. Similarly, when comparing CPSS risk for neurodevelopmental disorders (Supplementary Table S4) and SSD, there were significant differences in variance (Kruskal–Wallis test; *p *< 0.001), especially between SSD and ADHD and between SSD and ASD (multiple comparison using Bonferroni's method; *p *< 0.001 and *p *= 0.004, respectively; Supplementary Figure S3).

**Table 1:** Mean CPSS risk% and mean logit(*p*) for the diagnostic categories^a^.

	CPSS risk % ± SD (SE)	Logit(*p*) ± SD (SE)
Diagnostic categories		
Neurodevelopmental disorders^b^, *n* = 223	45.98 ± 41.38 (2.77)	−0.40 ± 4.13 (0.27)
Schizophrenia spectrum disorders^c^, *n* = 11	94.59 ± 14.85 (4.47)	4.64 ± 1.93 (0.58)
Depressive disorders, *n* = 58	65.89 ± 34.46 (4.52)	1.42 ± 3.36 (0.44)
Anxiety disorders (including OCD), *n* = 16	67.37 ± 32.50 (8.12)	1.22 ± 3.18 (0.79)
Somatic symptom disorders, *n* = 11	67.35 ± 35.32 (10.65)	1.11 ± 3.24 (0.97)
Others, *n* = 17	35.55 ± 40.49 (9.82)	−1.40 ± 3.81 (0.92)
Normal control group^d^		
	49.9 ± 35.1 (4.78)	−2.60 ± 2.57 (0.18)

^a^ Mean ± SDs shown unless otherwise stated.

^b^ These consist of Autism Spectrum Disorder (*n* = 141), Attention Deficit/Hyperactivity Disorder (*n* = 67), others (*n* = 15). Eleven of them have mild intellectual disabilities.

^c^ It consists of schizophreniform disorder (*n* = 2), brief psychotic disorder (*n* = 1), delusional disorder (*n* = 1), and schizophrenia (*n* = 7).

^d^ The mean CPSS risk% and mean logit(*p*) for the normal control group are sourced from our previous retrospective study (30). CPSS, child psychosis-risk screening system.

### 3.3. Predictive ability and cutoff values of the CPSS

**Figure 2:**
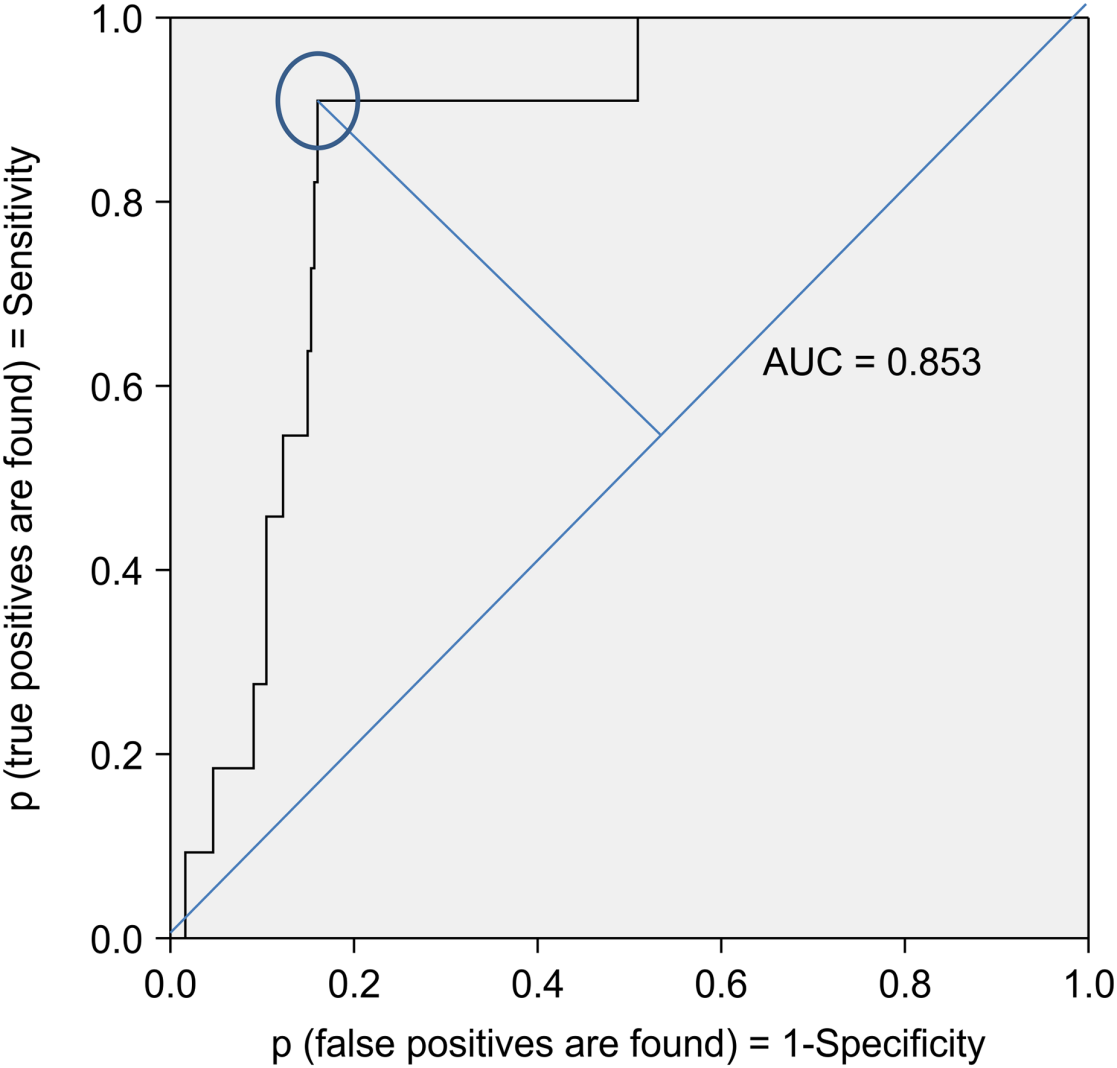
ROC curve for binary classification of the CPSS algorithm. The ROC curve showed that CPSS had an AUC of 0.853 (95% CI: 0.774–0.931). The cutoff values for CPSS risk% and logit(*p*) were determined to be 98.1% and 3.94, respectively, achieving the best mean of the sum of sensitivity (90.9%) and specificity (84.0%). ROC, receiver operating characteristic; AUC, area under the curve; CPSS, child psychosis-risk screening system.

The ROC curve showed that the CPSS had an AUC of 0.853 (95% CI: 0.774–0.931), indicating sufficient accuracy. The cutoff values for CPSS risk% and logit(*p*) were determined to be 98.1% and 3.94, respectively, achieving the best mean of the sum of sensitivity (90.9%) and specificity (84.0%) (Figure 2). Incidentally, ROC analysis using only the CBCL's thought problem subscale *t*-score (37)—which is often used to identify psychiatric symptoms in children—and the CBCL total *t*-score showed low discriminative power, with AUCs of 0.743 (95% CI: 0.650–0.881) and 0.588 (95% CI: 0.412–0.762), respectively. Of the participants, 18.3% (12.5% in pediatrics and 29.1% in psychiatry) were identified as those “at risk,” as they had values above the cutoff value of the CPSS. Moreover, 15.5% of the patients in the physical complaint group and 19.0% of the patients in the non-physical complaint group were classified as “at risk”.

### 3.4. Factors associated with the risk group identified using the CPSS

The results of the linear regression procedure showed no multicollinearity in the covariates, with VIF ranging from 1.129 to 2.034. All covariates were therefore used in logistic regression analysis. The results of cross-sectional logistic regression analysis using clinical data as the covariate and the CPSS risk group (group exceeding the CPSS cutoff value) as the dependent variable are shown in Table 2. Analysis showed that in addition to “schizophrenia spectrum diagnosis,” “winter birth” and “maltreatment” were factors associated with the risk group, with odds ratios of 38.05 (95% CI: 4.09–354.04; *p *= 0.001), 2.30 (95% CI: 1.17–4.53; *p *= 0.016), and 0.12 (95% CI: 0.02–0.76; *p *= 0.024), respectively.

**Table 2:** Cross-sectional logistic regression analysis predicting a participant's membership of the CPSS risk group (and thus being at high risk of developing psychosis in the future)^a^,^b^.

	*B*	SE	*p*	OR	95% CI for OR	
					Lower	Upper
Sex (male = 0, female = 1)	.387	.367	.292	1.473	.717	3.025
Age	.107	.060	.072	1.113	.990	1.251
Winter birth	.835	.345	.016*	2.304	1.171	4.533
Withdrawal	.243	.185	.189	1.275	.887	1.833
Bullying	−.018	.241	.941	.982	.613	1.575
Maltreatment	−2.057	.914	.024*	.128	.021	.767
Physical chief complaint^c^	−.541	.460	.240	.582	.236	1.434
Neurodevelopmental disorders	−.024	.479	.960	.976	.382	2.495
SSDs	3.639	1.138	.001**	38.052	4.090	354.041
Depressive disorders	−.129	.517	.804	.879	.319	2.424
Anxiety disorders	.683	.651	.295	1.979	.552	7.095
Somatic symptom disorders	−1.045	.832	.209	.352	.069	1.796

CPSS, Child Psychosis-risk Screening System; SSDs, schizophrenia spectrum disorders.

^a^ Logistic regression model statistics: Cox–Snell *R*^2 ^= 0.163, Nagelkerke *R*^2 ^= 0.265. The goodness-of-fit test of Hosmer and Lemeshow: *χ*^2 ^= 6.100, df = 8, *p *= 0.636. Discrimination accuracy = 84.2%.

^b^ Duplicate diagnoses are present.

^c^ This variable represents the main complaint of a participant being physical (and not non-physical) in nature.

* *p *< 0.05.

** *p *< 0.01.

The factors of sex, age, and whether the chief complaint was physical or non-physical were unrelated to the risk group.

## 4. Discussion

### 4.1. Clinical application of the CPSS in pediatric clinical practice

ROC analysis, with SSD onset as the outcome, revealed an AUC of 0.853, indicating that the CPSS has sufficient diagnostic discriminative ability. The results of this study suggest that the CPSS can be applied not only for psychosis risk identification, as indicated in our previous studies, but also for early detection of psychosis and differentiation of neurodevelopmental disorders. Considering the statistical treatment of future research data, an index that follows a normal distribution—*z* = logit(*p*)—seems to be appropriate as a risk index for CPSS. The cutoff point was *z *= 3.94, at which the sensitivity and specificity were 90.9% and 84.0%, respectively. The CPSS is a simple tool that allows the use of CBCL data (*t*-scores), which are widely used in child clinical practice. The clarification of the cutoff point for the risk index in this study indicates that the CPSS may be widely implemented in pediatric practices, even those unfamiliar with psychiatric care.

Of the pediatric patients, 12.5% were at risk of exceeding the CPSS cutoff point; the physical chief complaint group had a 15.5% risk. This is consistent with our clinical experience that a history of long pediatric attendance often precedes the initial visit of adolescent patients with SSD (38). When premorbid adjustment (PMA), defined as the level of functioning before the onset (23), declines and psychiatric symptoms are not prominent, the patient tends to be seen first in a general pediatrician's office. Subclinical psychotic experiences in children have also been reported to be significantly associated with functional somatic symptoms (24). If CPSS can be used to screen for psychosis risk in prolonged maladjustment groups such as truancy, hikikomori (39, 40), and groups with physical indeterminate complaints, prevention of the onset of psychosis and early intervention may be possible.

For early intervention, randomized controlled trials suggest that cognitive remediation therapy and fish oil (*ω*-3 fatty acids) supplementation improve cognition, symptoms, and function (41). Currently, the use of existing antipsychotic medications for the prodromal phase is not recommended except in limited circumstances (42, 43); therefore, the results of further interventional trials with non-invasive supplementations are awaited. The CPSS developed by us will be useful in selecting child participants for these interventional trials.

### 4.2. Differentiation between the psychosis risk group and those with neurodevelopmental disorders

As the potential for early screening grows, the need for discussion and effectiveness testing of specific interventions for different risk groups also grows. We found that SSD and neurodevelopmental disorders can be discriminated with sufficient accuracy considering CPSS risk, which can be easily measured in the clinical setting, can be combined with other test items (such as cognitive tests, neuropsychological tests, inflammatory markers, brain morphological imaging) (15, 41, 44, 45) and can be used for clinical diagnosis in the future. Differentiating children with psychosis risk from those with developmental disorders in childhood is difficult and is one of the most important challenges in child psychiatry. It has also been reported that a high rate of co-occurring neurodevelopmental disorders, such as ASD and ADHD, predates the onset of SSD, and the notion of a pan-developmental disturbance is gaining support (46). However, in the present study, our results indicate that CPSS, an algorithm incorporating the childhood psycho-behavioral characteristic patterns of schizophrenia, may discriminate early psychosis from ASD and ADHD with sufficient accuracy. Even if SSD is incorporated into the neurodevelopmental disorder hypothesis, its patterns of childhood subclinical psycho-behavioral characteristics would be quite different from those of other developmental disorders. This study focused on withdrawal and lack of aggression as characteristics of children in the pre-psychotic phase, based on our previous study that found the combination of childhood withdrawal, thinking problems, and lack of aggressive behavior could predict the development of schizophrenia (30). In the context of schizophrenia, the low aggression that has been observed in childhood (29, 47) appears to be frequently reversed in adolescence (48). There is also evidence that a pattern of less withdrawal and more aggression in childhood is a predictor of non-psychosis (5). Notably, lack of appropriate aggression may increase interpersonal stress and decrease adjustment; on the other hand, neurodevelopmental disorders are not marked by low aggression in childhood. Furthermore, CPSS risk for neurodevelopmental disorders is more varied than for other psychiatric disorders, and there are a small number of CPSS high-risk patients among the ASD and ADHD groups whose conditions exceed the cutoff value. Future longitudinal studies should clarify whether these patients happen to have neurodevelopmental disorders with psychotic risk or whether they are pre-psychotic cases with a temporary phenotype of neurodevelopmental disorders in the pathway before onset. There is an important clinical rationale for differentiation between SSD and neurodevelopmental disorders. Although various special education plans have been designed for neurodevelopmental disorders, children in the psychosis risk group would benefit more from medical cognitive remediation therapy to promote appropriate aggression expression (for example, being able to say “no” and communicate anger) prior to such educational promotion and inclusion.

### 4.3. Winter birth and abuse: factors associated with the risk group

Winter birth and abuse were identified as factors associated with the risk group identified using the CPSS. Winter birth has already been reported as a risk factor for schizophrenia (6, 49), consistent with previous evidence. This factor may be associated with the development of vulnerability in SSD, as previous studies have indicated (50). Although abuse is considered a risk factor in the multi-domain model presented by previous studies (5, 6), the present study showed a negative correlation with an OR of 0.128. The first possible explanation for the negative correlation between abuse and SSD risk is the lack of aggression, a subclinical feature of premorbid childhood (29, 30). This may be a genetic aspect from a parent with low aggression, and the low aggression of both parent and child may be inhibitory to the occurrence of abuse. The second is that abuse triggers the onset of psychosis but plays little role in the vulnerability itself. The two-hit model (51), now the dominant hypothesis for schizophrenia, suggests that early perinatal insults (genetic background and/or environmental factors) lead to dysfunction of neuronal circuits and vulnerability to the disease, while a second “hit” (such as psychosocial stress) during a critical brain development period in adolescence may trigger onset of the disease by acting on existing vulnerabilities. As aforementioned, the CPSS was developed not as an SSD symptom detection tool but only to detect premorbid signs in childhood, i.e., a vulnerability assessment tool (30). It is difficult to differentiate between the trauma-related disorders and psychosis risk groups in childhood PLE cases (52, 53). However, in the present study, the CPSS risk for PTSDs was found to be remarkably low at *z *= −3.70 ± 2.98 (Supplementary Table S3), suggesting that the CPSS is a useful adjunctive diagnostic tool in differentiating these two groups of disorders. However, some reports suggest that the trauma of abuse can alter the structural and functional levels of the brain through epigenetic mechanisms and contribute to vulnerability itself (54); thus, the relationship between trauma and vulnerability formation will require careful discussion in the future.

A limitation of the present study is the small sample size; only 11 patients with SSDs were recruited. Furthermore, this is a cross-sectional study in which outcomes were assessed based on current SSD diagnosis. Future longitudinal studies with larger sample sizes are needed to ascertain whether children in the risk group have a higher incidence of the disease. CPSS risk was calculated from the scores of the CBCL questionnaire, which was answered by the patients’ parent(s). Psychological diagnoses were not obtained for the parents and neither were data on family structure. Therefore, bias by the respondent may have been incorporated, with significant ramifications for reliability. We used our original ad-hoc scales for maltreatment, bullying, and withdrawal, which is also a limitation of our study.

In the future, research with larger cohorts is needed to test the validity of the CPSS algorithm and its utility as a screening tool. The clinical application of screening tools will need to be carefully considered in further research, especially in conjunction with their risks and benefits for early intervention strategies. The cost-effectiveness of screening and intervention plans based on predictive childhood characteristics is significant (55). Moreover, identifying the risk group in childhood would promote longer-term studies that reveal the dynamic system from vulnerability to symptom manifestation.

In conclusion, the results of this study provide preliminary evidence that the CPSS could be applied in pediatric clinical practice not only for psychosis risk identification but also for early detection of psychosis and differentiation from neurodevelopmental disorders. Furthermore, the identification of risk groups based on children's subclinical characteristics may allow for earlier preventive interventions in the critical period of psychosis. However, the sample size of this study was small and further validation of the results in larger studies ideally with longitudinal design are required prior to recommendation of use in routine clinical practice.

## Data Availability

The raw data supporting the conclusions of this article will be made available by the authors, without undue reservation. The Child Psychosis-risk Screening System (CPSS) is available in an interactive web system at https://nakayama-lab.japanwest.cloudapp.azure.com/CPSS6-18/.
