# Supplementary material for: Diagnostic specificity of the child psychosis-risk screening system with a focus on the differentiation of schizophrenia spectrum disorders and neurodevelopmental disorders
Source: Front Child Adolesc Psychiatry. 2023 Aug 4;2:1230346. doi: 10.3389/frcha.2023.1230346 (PMC11732021; doi:10.3389/frcha.2023.1230346)
Supplement: Supplementary file 1 [file Datasheet1.docx]

***Supplementary Material***

**Diagnostic Specificity of the Child Psychosis-risk Screening System with a Focus on the Differentiation of Schizophrenia Spectrum Disorders and neurodevelopmental Disorders**

**Yukiko Hamasaki^*^, Yuko Sakaue, Masahiro Matsuo, Riku Sanada, Takao Nakayama, Shugo Michikoshi, Satoko Ueba, Naoki Kurimoto, Takatoshi Hikida, Toshiya Murai**

***Correspondence:** Yukiko Hamasaki: hamasaki@kyoto-wu.ac.jp

**1 Supplementary Table S1.** Demographic characteristics of patients

|  | **n=336** |  |
| --- | --- | --- |
| Sex (Male/Female) | 212/124  63.1% male |  |
| Age (in years)^a^ | 11.15±3.37 |  |
| Pediatric/Psychiatric patients | 216/120 |  |

^a^ Mean±SDs shown unless otherwise stated.

**2 Supplementary Table S2.** Demographic characteristics, diagnosis, and chief complaints of pediatric and psychiatric patients

|  | **Pediatric patients**  n=216 | **Psychiatric patients**  n=120 |
| --- | --- | --- |
| Sex (Male/Female) | 164/52  75.9% male | 48/72  40.0% male |
| Age (in years)^a^ | 9.63±2.69 | 13.87±2.68 |
| **Diagnosis ^b^** | **n** | **n** |
| Neurodevelopmental disorders | 197 | 39 |
| Schizophrenia spectrum disorders^c^ | 0 | 11 |
| Bipolar disorders | 0 | 1 |
| Depressive disorders | 6 | 68 |
| Anxiety disorders | 8 | 6 |
| Obsessive-compulsive disorders | 8 | 6 |
| Posttraumatic stress disorder | 3 | 0 |
| Somatic symptom disorders | 16 | 8 |
| Physical disorders | 24 | 0 |
| Epilepsy | 5 | 3 |
| Others | 23 | 15 |
| **Chief complaints** | **n** | **n** |
| Physical/non-physical | 24/192 | 34/86 |

^a^ Mean±SDs shown unless otherwise stated. ^b^ Duplicate diagnoses are present. ^C^ These consist of schizophreniform disorder (n=2), brief psychotic disorder (n=1), delusional disorder (n=1), and schizophrenia (n=7)

**3** **Supplementary Table S3.** Mean CPSS risk % and mean logit(p) for each diagnosis and chief complaint ^a^

| Diagnosis^b^ |  |  |
| --- | --- | --- |
|  | Mean CPSS risk %SD (SE) | Mean Logit(p)±SD (SE) |
| Neurodevelopmental disorders, n=236 | 47.16±41.41 (2.69) | -0.33±4.10 (0.26) |
| Schizophrenia spectrum disorders^c^, n=11 | 94.59±14.85 (4.47) | 4.64±1.93 (0.58) |
| Bipolar disorders, n=1 | 87.15 | 1.915 |
| Depressive disorders, n=74 | 66.31±34.53 (4.01) | 1.38±3.32 (0.38) |
| Anxiety disorders, n=14 | 65.23±38.96 (2.26) | 1.01±3.96 (1.05) |
| Obsessive-compulsive disorders, n=14 | 66.75±38.52 (10.29) | 1.59±3.56 (0.95) |
| Posttraumatic stress disorder, n=8 | 13.88±31.69 (11.20) | -3.70±2.98 (1.05) |
| Somatic symptom disorders, n=24 | 58.65±38.02 (7.76) | 0.46±3.00 (0.61) |
| Physical disorders, n=24 | 46.86±39.74 (8.11) | -0.47±3.27 (0.66) |
| Epilepsy, n=8 | 47.84±39.29 (13.89) | -0.31±2.94 (1.04) |
| Eating disorder, n=8 | 49.37±37.37 (13.21) | -0.36±2.99 (1.05) |
| Others, n=28 | 37.08±39.23 (7.41) | -1.44±3.79 (0.71) |
| Chief complaints |  |  |
| Physical/non-physical, n=58/278 | 54.86±37.84 (4.96)/ 51.65±41.30 (2.47) | 0.33±3.54 (0.46)/ 0.11±4.11 (0.24) ^c^ |

^a^ Mean±SDs shown unless otherwise stated. ^b^ Duplicate diagnoses are present. ^C^ It consists of schizophreniform disorder (n=2), brief psychotic disorder (n=1), delusional disorder (n=1), and schizophrenia (n=7). ^c^ *t*-test comparison. *t*=-0.365, *p*=0.715. CPSS, Child Psychosis-risk Screening System

**4 Supplementary Table S4.** Mean CPSS risk % and mean logit(p) by main diagnosis of neurodevelopmental disorders ^a,b^

|  | | CPSS risk %±SD (SE) | Logit(p)±SD (SE) |
| --- | --- | --- | --- |
| Diagnosis |  | |  |
| Autism spectrum disorder (n=141) | | 54.61±41.61 (3.50) | 0.41±4.22 (0.35) |
| Attention deficit/hyperactivity disorder (n=67) | | 30.12±36.54 (4.46) | -2.09±3.60 (0.44) |
| Others (n=15) | | 35.75±38.33 (9.89) | -0.50±3.11 (0.80) |

^a^ Mean±SDs shown unless otherwise stated. ^b^ Eleven of them have mild intellectual disabilities. CPSS, Child Psychosis-Risk Screening System; SD, standard deviation

**5 Supplementary Figure S1.** Distribution of CPSS risk %


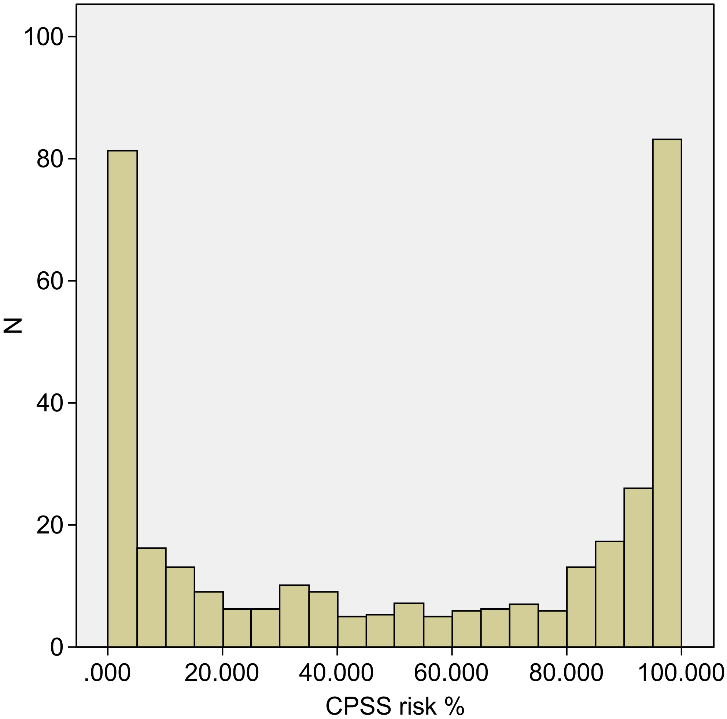


Patients’ risk of psychosis was identified. CPSS, Child Psychosis-Risk Screening System. Mean CPSS risk %=52.20±40.69 (2.22)

**6 Supplementary Figure S2.** Distribution of CPSS logit(p)


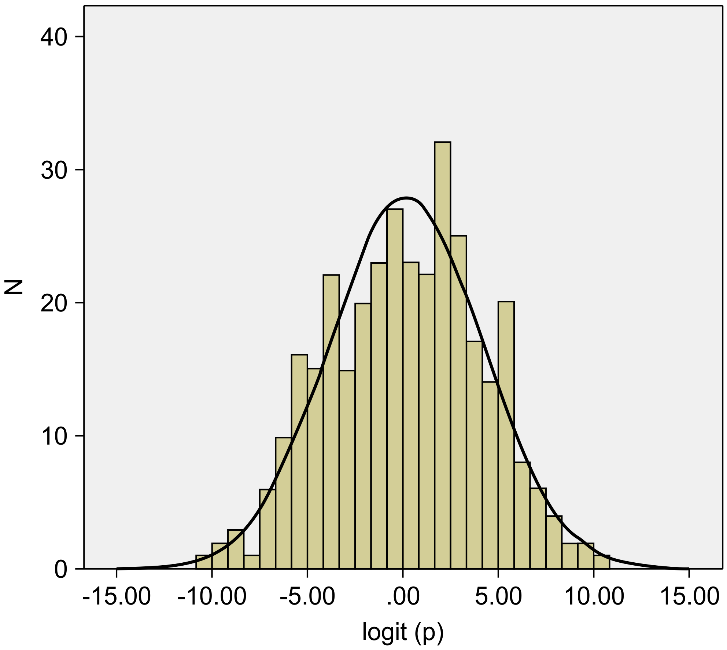


Mean CPSS logit(p)=0.15±4.02 (0.21)

Shapiro–Wilk test determined (p)=0.429. CPSS logit(p) follows a normal distribution.

CPSS, Child Psychosis-Risk Screening System

**7 Supplementary Figure S3.** Analysis of variance of using CPSS for neurodevelopmental disorders (ASD, ADHD, and others) and SSD: Box-plots of the logit(p)


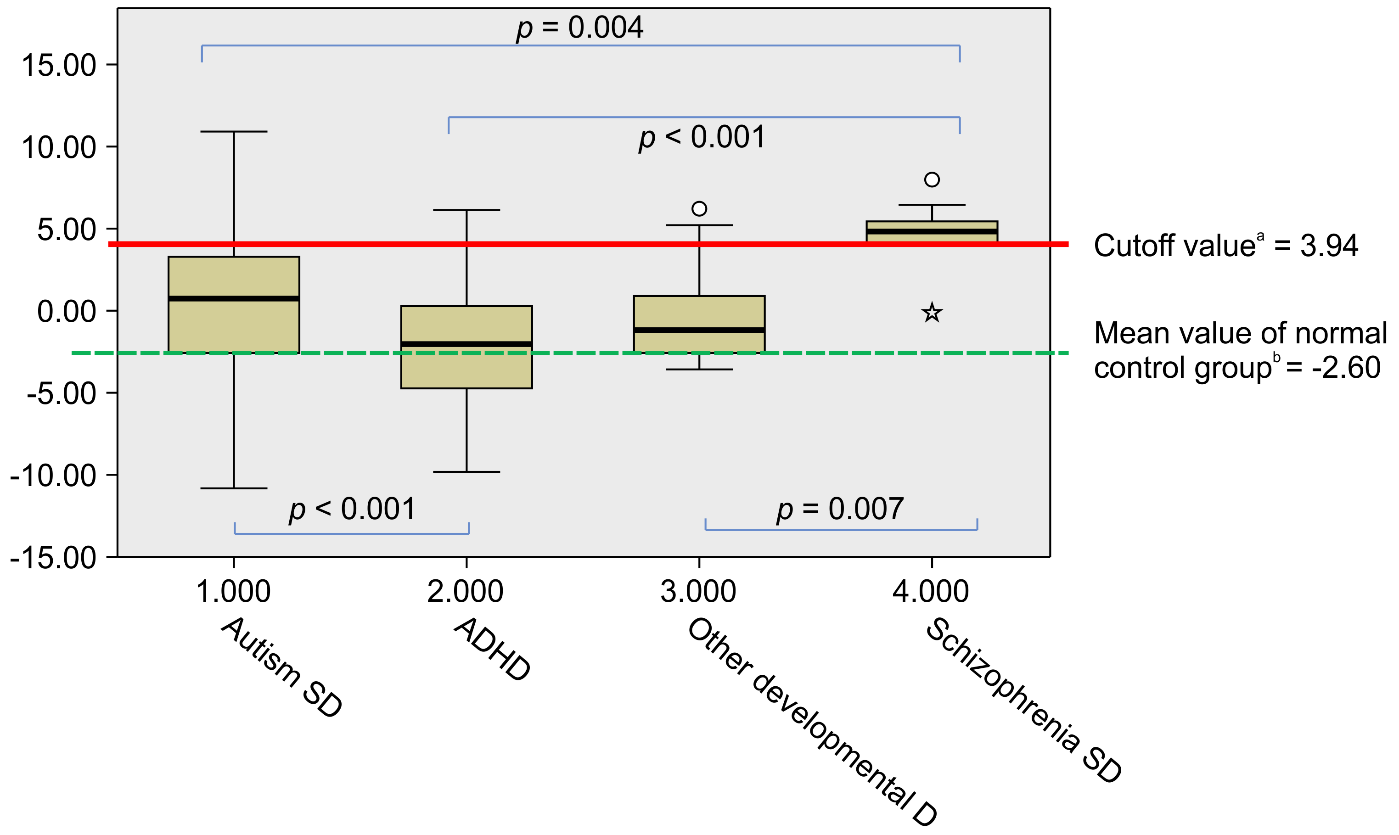


^a^ The ROC curve analysis resulted in a cutoff value of 3.94 for logit(p) (sensitivity: 90.9% and specificity: 84.0%). ^b^ The mean logit(p) for the normal control group is sourced from our previous retrospective study (30).

There was a difference in logit(p) among the four diagnostic categories (Kruskal–Wallis test;　*p*<0.001). Compared with patients with ADHD and ASD, patients with SSD had significantly elevated logit(p) (*p*<0.001 and *p*=0.004, respectively, for multiple comparisons using the Bonferroni method).

CPSS, Child Psychosis-Risk Screening System; ASD, autism spectrum disorder; ADHD, attention deficit hyperactivity disorder; SSD, schizophrenia spectrum disorders; ROC curve, receiver operating characteristic curve

**8 Supplementary Appendix S1**

**Logistic regression model as the algorithm for identifying psychosis risk in children** **(for CBCL/4–18)**

Risk indicators: p ^a^, z ^b^, risk % ^c^

1. p=$\frac{1}{1+exp(-z)}$
2. z = -6.517+0.119×Ti-0.066×Tii+0.031×Tiii+0.035×Tiv+0.279×Tv+0.129×Tvi-0.011×Tvii-0.428×Tviii = logit(p)
3. risk % =p×100

Child Behavior Checklist (CBCL)/4-18 syndrome subscale t-scores

Ti: Withdrawn

Tii: Somatic complaints

Tiii: Anxious/Depressed

Tiv: Social problems

Tv: Thought problems

Tvi: Attention problem

Tvii: Delinquent behavior

Tviii: Aggressive behavior

**9 Supplementary Appendix S2**

**The algorithm of Child Psychosis-Risk Screening System (CPSS) using CBCL/6-18**

Risk indicators: p ^a^, z ^b^, risk % ^c^

1. p=$\frac{1}{1+exp(-z)}$
2. z = -6.517+0.119×Tii-0.066×Tiii+0.031×Ti+0.035×Tiv+0.279×Tv+0.129×Tvi-0.011×Tvii-0.428×Tviii = logit(p)
3. p×100=risk %

Child Behavior Checklist (CBCL)/6-18 syndrome subscales

Ti: Anxious/Depressed

Tii: Withdrawn/Depressed

Tiii: Somatic Complaints

Tiv: Social Problems

Tv: Thought Problems

Tvi: Attention Problems

Tvii: Rule-Breaking Behavior

Tviii: Aggressive Behavior
